# The Nutrient-Responsive Molecular Chaperone Hsp90 Supports Growth and Development in *Drosophila*

**DOI:** 10.3389/fphys.2021.690564

**Published:** 2021-06-22

**Authors:** Yuya Ohhara, Genki Hoshino, Kyosuke Imahori, Tomoya Matsuyuki, Kimiko Yamakawa-Kobayashi

**Affiliations:** ^1^School of Food and Nutritional Sciences, University of Shizuoka, Shizuoka, Japan; ^2^Graduate School of Integrated Pharmaceutical and Nutritional Sciences, University of Shizuoka, Shizuoka, Japan; ^3^Life Science Center for Survival Dynamics, Tsukuba Advanced Research Alliance (TARA), University of Tsukuba, Tsukuba, Japan

**Keywords:** heat shock protein 90, fat body, insulin-like peptide, target of rapamycin, *Drosophila*

## Abstract

Animals can sense internal nutrients, such as amino acids/proteins, and are able to modify their developmental programs in accordance with their nutrient status. In the fruit fly, *Drosophila melanogaster*, amino acid/protein is sensed by the fat body, an insect adipose tissue, through a nutrient sensor, target of rapamycin (TOR) complex 1 (TORC1). TORC1 promotes the secretion of various peptide hormones from the fat body in an amino acid/protein-dependent manner. Fat-body-derived peptide hormones stimulate the release of insulin-like peptides, which are essential growth-promoting anabolic hormones, from neuroendocrine cells called insulin-producing cells (IPCs). Although the importance of TORC1 and the fat body-IPC axis has been elucidated, the mechanism by which TORC1 regulates the expression of insulinotropic signal peptides remains unclear. Here, we show that an evolutionarily conserved molecular chaperone, heat shock protein 90 (Hsp90), promotes the expression of insulinotropic signal peptides. Fat-body-selective *Hsp90* knockdown caused the transcriptional downregulation of insulinotropic signal peptides. IPC activity and systemic growth were also impaired in fat-body-selective *Hsp90* knockdown animals. Furthermore, *Hsp90* expression depended on protein/amino acid availability and TORC1 signaling. These results strongly suggest that *Hsp90* serves as a nutrient-responsive gene that upregulates the fat body-IPC axis and systemic growth. We propose that *Hsp90* is induced in a nutrient-dependent manner to support anabolic metabolism during the juvenile growth period.

## Introduction

Nutrient-dependent anabolic metabolism and growth are fundamental physiological processes in juvenile development. In vertebrates and invertebrates, this process is supported by nutrient-responsive anabolic hormones. Members of the insulin-like peptide (ILP) family, including insulin-like growth factors in vertebrates and ILPs in insects, play an essential role in nutrient-dependent anabolism and systemic growth (Garofalo, 2002; Hietakangas and Cohen, 2009; Tennessen and Thummel, 2011; Okamoto and Yamanaka, 2015). ILPs are secreted from specific endocrine organs to promote various cellular events, including protein synthesis, cell growth, and proliferation. In the fruit fly, *Drosophila melanogaster*, eight ILPs are encoded in the genome; among them, ILP2, ILP3, and ILP5 are mainly synthesized in the neuroendocrine cells called insulin-producing cells (IPCs) (Ikeya et al., 2002; Okamoto and Yamanaka, 2015). ILPs secreted from IPCs promote insulin signaling in the peripheral organs and induce subsequent anabolic metabolism and growth. ILP production in IPCs is dependent on nutrient status, and the most crucial nutrient cue for ILP production is protein/amino acids. If the amino acid/protein content is low in a culture medium, ILP secretion is blocked, leading to the downregulation of systemic insulin signaling and growth (Géminard et al., 2009; Okamoto and Yamanaka, 2015).

Amino acid/protein availability is mainly sensed by insect adipose tissue, termed “fat body.” The fat body synthesizes triacylglycerol (TAG) which is supplied to other tissues during development and senses amino acids through an evolutionarily conserved nutrient sensor, the target of rapamycin (TOR) (Colombani et al., 2003; Layalle et al., 2008). TOR forms a complex called TOR complex 1 (TORC1), which consists of TOR, a regulatory protein associated with mTOR (Raptor), and lethal with Sec13 protein 8 (Lst8). TORC1 activity is upregulated by amino acids, including leucine and arginine (Saxton and Sabatini, 2017). In the *Drosophila* fat body, the arginine transporter SlimFast is required for TORC1 activation (Colombani et al., 2003; Layalle et al., 2008). TORC1 activation leads to fat body-derived secretion of peptides, including stunted and growth-blocking peptides (GBP)1 and GBP2, into the hemolymph (Delanoue et al., 2016; Koyama and Mirth, 2016; Meschi et al., 2019). The fat body also secretes a small peptide called CCHamide-2 (CCHa2) in response to glucose and protein feeding (Sano et al., 2015). These insulinotropic signal peptides are received by their receptors expressed in IPCs or IPC-connecting neurons. Stunted and CCHa2 activate their specific receptors expressed in the IPCs to promote ILP release (Sano et al., 2015; Delanoue et al., 2016), whereas GBP1 and GBP2 act on the IPC-connecting neurons to alleviate the inhibition exerted by the IPC-connecting neurons on the IPCs (Meschi et al., 2019). In contrast, under amino acid/protein starvation, the fat body releases a signal peptide called Eiger to limit ILP expression in the IPCs (Agrawal et al., 2016). Although Eiger is the tumor necrosis factor (TNF) superfamily ligand, which was originally identified as a cell-death-inducing signal peptide, fat body-derived Eiger functions as an insulinostatic signal peptide. In brief, downregulation of TORC1 causes transcriptional upregulation of a metalloprotease, TNF-α-converting enzyme (TACE), and TACE cleaves the extracellular domain of Eiger (Agrawal et al., 2016), which then acts on IPCs to suppress ILP production (Agrawal et al., 2016). Furthermore, amino acids are sensed by glial cells, and the downstream cholinergic neurons promote *ILP5* gene expression in the IPCs (Okamoto and Nishimura, 2015). Additionally, leucine acts on the IPCs directly through the amino acid transporters Juvenile hormone Inducible-21 and Minidiscs to stimulate ILP secretion (Manière et al., 2016; Ziegler et al., 2018).

Although the importance of TORC1 and downstream insulinotropic/insulinostatic signal peptides has been elucidated, the mechanism by which TORC1 regulates insulinotropic/insulinostatic signal peptide production in the fat body remains unclear. In addition, while the fat body is also known as the center of TAG synthesis and storage, how the fat body coordinately regulates TAG metabolism and insulinotropic/insulinostatic peptide production is unknown. Therefore, identifying these mechanisms is key to a better understanding of juvenile development. Here, we hypothesized that inhibition of these mechanisms in the fat body might cause developmental arrest at the larval stage. To explore the hypothesis, we performed fat-body-selective RNAi screening to select genes that are essential for larval development. Two rounds of RNAi screening narrowed down the list of hits and identified five essential genes. We further investigated the roles of these genes in TAG levels and growth. The genes did not positively regulate TAG levels in the larval stage. However, an evolutionarily conserved chaperone, heat shock protein 90 (Hsp90), was demonstrated to be essential for the progression of larval growth. Our data indicate that adipocyte *Hsp90* is required for the upregulation of insulinotropic peptide expression, IPC activation, and systemic growth. Furthermore, *Hsp90* expression was dependent on protein/amino acid feeding and TORC1 signaling. The collective data provide a molecular basis for understanding of how the fat body couples nutrient status to insulinotropic endocrine signaling.

## Materials and Methods

### *Drosophila* Stocks and Medium

The fly stocks used in this study are listed in Supplementary Table 1. *Oregon R* was used as the wild-type (WT) strain. The RNAi stocks used for RNAi screens are listed in Supplementary Tables 2, 3. Fly stocks were reared on a glucose/cornmeal/yeast medium (1 g glucose, 0.7 g cornmeal, 0.4 g yeast extract, 50 mg agar in 10 mL water) supplemented with 30 μL propionic acid and 35 μL butylparaben (167 mg/mL in 70% ethanol). Flies were maintained at 25°C under a 12-hour light/dark cycle.

A nutrient-rich semi-defined medium, German food, was used for all experiments, except for an RNAi screen. Briefly, 22.5 g German food powder (Genesee Scientific, San Diego, CA, United States, 66-115) was added to 100 mL water, and the mixture was boiled using a microwave. After stirring for 30 min at room temperature (18–25°C), 600 μL propionic acid was added to the mixture to obtain the German food medium.

To investigate the effect of nutrients on gene expression, we used a series of limited culture media, including control, starvation, and protein-deficient media. Supplementary Table 4 shows the nutrient composition of each of these media. The composition was determined by referring to a defined medium (called “Medium C” in the original paper) (Sang, 1956). Ingredients other than vitamin mix and propionic acid were added to water and boiled using a microwave oven. After stirring for 30 min at room temperature, propionic acid and vitamin mix were added to the mixture.

### Fat Body-Selective RNAi Screening

Fat body-selective RNAi screening was performed to identify novel regulators of larval development. In the first RNAi screen, we screened 231 genes that were previously identified as TAG metabolism-related genes (Pospisilik et al., 2010; Supplementary Table 2). The second RNAi screen was performed to confirm whether the developmental defects observed in the first screen were reproducible using 2–3 independent RNAi strains (Supplementary Table 3). In the first and the second RNAi screens, virgins carrying homozygous *Cg-Gal4* were crossed with *UAS-RNAi* males to obtain the offspring in which a target gene was knocked down in the fat body (*Cg-Gal4* > *UAS-RNAi against gene-of-interest*). To obtain control animals (*Cg-Gal4* > +), *Cg-Gal4* females and *w*^1118^ males were crossed. Parent flies were cultured in a plastic vial with a glucose/cornmeal/yeast medium for 2 days, and the developmental phenotypes of their progenies were observed to identify knockdown strains showing developmental arrest.

### Staging, Nutrient Manipulations, and Analysis of Body Weight and Developmental Progression

Parent flies were maintained in plastic bottles (Genesee Scientific, San Diego, CA, United States, 32-310) and allowed to lay eggs for 24 h on grape juice agar plates (2 g agar in 100 mL grape juice, poured into a 4.5 cm × 1.6 cm plastic dish) supplemented with dry yeast powder (Oriental Yeast Co., Ltd., Japan). Newly hatched larvae were transferred to vials containing German food medium. The larvae were cultured at 25°C unless stated otherwise under a 12-hour light/dark cycle for defined times. Nutritional manipulations were performed using a series of nutrient limited culture media (see section “*Drosophila* Stocks and Medium” and Supplementary Table 4). Larvae cultured on the German food medium were transferred to a non-nutritive starvation medium at 24 h after hatching (hAH). After a further 24 h, the larvae were transferred to a nutrient-enriched control or a protein-deficient medium and cultured for defined times.

To measure body weight, larvae were washed with distilled water, blotted on KimWipes (Crecia, Japan) to remove distilled water, and collected in 1.5 mL plastic tubes before weighing. Larvae were weighed in more than three batches of 10–15 larvae in each experimental group. The body weight of individual larva was then calculated for each batch.

The same egg collection and larval rearing methods were used to analyze the developmental progression. Developmental stages and lethality were scored every 24 h.

### Lipid Extraction and TAG Level Measurement

To investigate TAG content in knockdown animals, lipid extraction and TAG level measurements were conducted as follows. Ten to 15 frozen larvae collected in 1.5 mL plastic tubes were homogenized in liquid nitrogen using plastic pestles, and 1 mL chloroform-methanol mixture (2:1, v/v) was added to the smashed samples. After overnight incubation at room temperature, the samples were centrifuged for 15 min at 3,800 × *g*, and the supernatants were collected into fresh tubes. The extracts were dried in a centrifugal evaporator (EYELA, Japan) and re-dissolved in 500 μL phosphate-buffered saline (PBS) with 0.1% (v/v) Tween20. TAG was quantified using the Triglyceride *E*-test Wako (Fujifilm, Japan, 290-63701) in accordance with the manufacturer’s protocol. TAG levels were normalized to the sample weight. The mean TAG level in the control at 72 hAH was set to 100, and the relative TAG level of each sample was calculated.

### Quantitative RT-PCR

Quantitative RT-PCR (qPCR) was performed to measure the expression levels of *Hsp90* and growth-regulating genes including insulinotropic/insulinostatic signal peptide-coding genes. Total RNA was extracted from larvae and fat bodies (5–6 pooled fat bodies) using TRIzol (Thermo Fisher Scientific, 15596026) and the RNeasy Micro kit (QIAGEN, 74004), respectively. Reverse transcription was performed using the SuperScript III kit (Thermo Fisher Scientific, 18080051), and the obtained cDNA was used as a template for qPCR using the Quantifast SYBR Green PCR kit (QIAGEN, 204056) and Rotor-Gene Q (QIAGEN). All reactions were performed at 95°C for 10 min, followed by 50 cycles of 95°C for 10 s and 60°C for 30 s. Dissociation curve analysis was applied to all reactions to ensure the presence of a single PCR product. The expression levels of the target genes were calculated using the relative standard curve method. Stock cDNA used for the relative standard curves was synthesized from pooled RNA derived from larvae bred under the same conditions and diluted serially. The expression levels of the target genes were normalized to an endogenous reference gene, ribosomal protein 49 (*rp49*) (also known as ribosomal protein L32), because *rp49* is stably expressed across various nutrient conditions (Ponton et al., 2011). The mean expression level of the control was set to 1. The primer sets used for qPCR are listed in Supplementary Table 5.

### Immunohistochemistry and Histochemistry

Immunostaining was performed to investigate the expression levels of ILP2 and phospho-S6. Larvae were dissected in PBS and fixed for 25 min with 4% paraformaldehyde in 0.1% PBT (0.1% Triton X-100 in PBS). Tissues were washed with 0.1% PBT three times for 10 min each, blocked with 2% goat serum (Sigma-Aldrich, G9023) in 0.1% PBT for 30 min, and then incubated at 4°C overnight with a primary antibody against ILP2 (rabbit IgG, polyclonal) (Okamoto et al., 2012) at a 1:500 dilution or against phospho-S6 (rabbit IgG, polyclonal) (Romero-Pozuelo et al., 2017) at a 1:1,000 dilution in a blocking solution. Tissues were washed with 0.1% PBT three times for 10 min each, and incubated at 4°C overnight with Alexa 488-conjugated goat IgG (Thermo Fisher Scientific, A11081) at 1:1,000 dilution and Hoechst 33342 (Thermo Fisher Scientific, 62249) at 1:1,500 dilution in 0.1% PBT. After washing with 0.1% PBT three times for 10 min each, brains and fat bodies were dissected out and mounted in a mounting medium [2.4 g Mowiol 4-88 (Sigma-Aldrich, 81381), 6 g glycerol, 6 mL distilled water, and 12 mL 0.2 M Tris–Cl (pH 8.5)].

To investigate the expression pattern of Hsp90 protein, expression of GFP-tagged Hsp90 was observed using histochemistry as follows: GFP-tagged Hsp90-expressing larvae cultured on a series of limited culture media were dissected in PBS and fixed for 25 min with 4% paraformaldehyde in 0.01% PBT. Tissues were washed with 0.01% PBT three times for 10 min each, and incubated at 4°C overnight with Hoechst 33342 at 1:1,500 dilution and Alexa 546-conjugated phalloidin (Thermo Fisher Scientific, A22283) at 1:500 dilution in 0.01% PBT. GFP-tagged Hsp90 was detected without signal enhancement by an antibody against GFP. After washing with 0.01% PBT three times for 10 min each, the fat bodies were mounted in the mounting medium.

Images were taken with a Zeiss LSM700, and image analyses were performed using Image J/Fiji software (Schindelin et al., 2009).

### Statistical Analysis

Statistical analyses were performed using R software (version 3.6.2)^1^ (R Core Team, 2019). Data were analyzed using Student’s *t*-test, Mann–Whitney *U*-test, Dunnett’s test, Tukey’s test, or Steel’s multiple comparison tests.

## Results

### Fat-Body-Selective RNAi Screen Identifies Novel Regulators of Growth and Development

**FIGURE 1:**
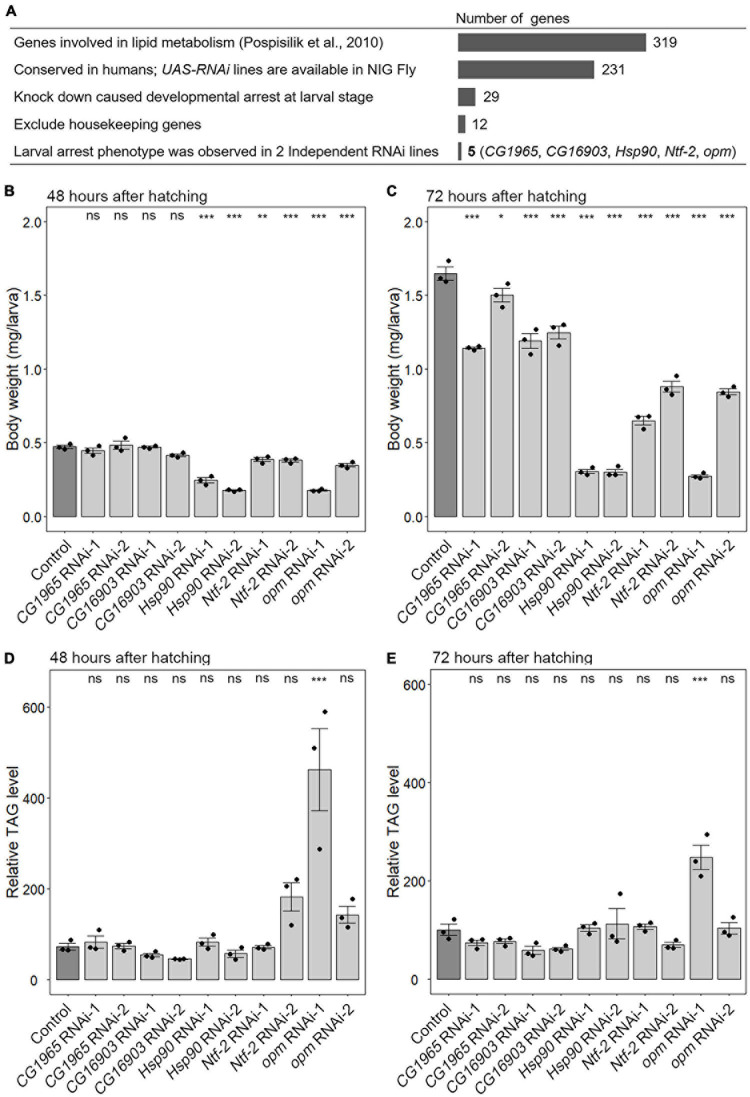
A fat-body-selective RNAi screen to identify novel growth regulators. **(A)** Scheme of a fat-body-selective RNAi screen to identify novel growth regulators. Candidate genes and the results are summarized in Supplementary Tables 1, 2. NIG Fly, Fly Stocks of National Institute for Basic Biology (Japan). **(B–E)** Fat-body-selective knockdown of *Hsp90*, *Ntf-2*, and *opm* causes growth retardation. The body weights **(B,C)** and the relative triacylglycerol (TAG) levels **(D,E)** in control (*Cg-Gal4* > +) (dark gray) and knockdown animals (*Cg-Gal4* > *UAS-RNAi against gene-of-interest*) (light gray) at 48 **(B,D)** and 72 h after hatching **(C,E)**. In all graphs, the average values of triplicate datasets are shown with SEs and scatter plots. Ten or 15 larvae were pooled in each sample. Different lowercase letters indicate statistically significant differences (*P < 0.05, **P < 0.01, ***P < 0.001; Dunnett’s test). ns, not significant.

A previous study conducted a genome-wide RNAi screen and identified 319 genes whose knockdown caused a decrease or increase in TAG levels in the adult stage (Pospisilik et al., 2010). Among these genes, we focused on 231 genes whose corresponding RNAi stocks were available in NIG Fly Stock Center Japan and whose orthologous genes exist in humans (Figure 1A). To knock down the candidate genes, fat-body-selective *Cg-Gal4* was used to overexpress RNAi constructs under the control of the upstream activating sequence (UAS) (*Cg-Gal4* > *UAS-RNAi against gene-of-interest*) (Asha et al., 2003). We found 29 genes whose knockdown caused developmental arrest at the larval stage (Supplementary Table 2 and Figure 1A). After excluding 17 housekeeping genes, including translation initiation complex subunits, amino acid-tRNA ligases, ribosome components and regulators, spliceosome subunits, and proteasome subunits (Supplementary Table 2), the resulting 12 genes were knocked down in the fat body using 2–3 RNAi lines to confirm that the larval-arrest phenotype was reproducible (Figure 1A). We found that the larval-arrest phenotype of *CG1965*, *CG16903*, *nuclear transport factor-2* (*Ntf-2*), *Hsp90*, and *opossum* (*opm*) was reproducible in knockdown animals (Supplementary Table 3 and Figure 1A). This indicates that these five genes are required for proper larval development.

Next, we measured the body weight and TAG content in the knockdown animals. The body weights of *CG1965* and *CG16903* RNAi animals were slightly reduced compared to that of the control (*Cg-Gal4* > +) at 72 hAH (Figures 1B,C), while the body weight of *Hsp90*, *Ntf-2*, and *opm* RNAi animals was more severely reduced at 48 and 72 hAH (Figures 1B,C). These results suggest that *Hsp90*, *Ntf-2*, and *opm* regulate systemic growth, whereas *CG1965* and *CG16903* are required for the onset of pupariation. Notably, although these genes were originally identified as the possible regulators of TAG metabolism in the adult stage (Pospisilik et al., 2010), the TAG levels were not significantly reduced in any knockdown larvae at 48 and 72 hAH (Figures 1D,E). This result suggests that these genes have little or no effect on TAG metabolism during larval development.

### *Hsp90* Is Required for IPC Activation and Larval Growth

In our RNAi screen, fat-body-selective *Hsp90* knockdown animals showed the most severe growth defect among the others (Figures 1B,C), suggesting that *Hsp90* expressed in the fat body plays an essential role in organismal growth. We subsequently focused on *Hsp90* and performed further genetic analyses to elucidate the *in vivo* functions.

**FIGURE 2:**
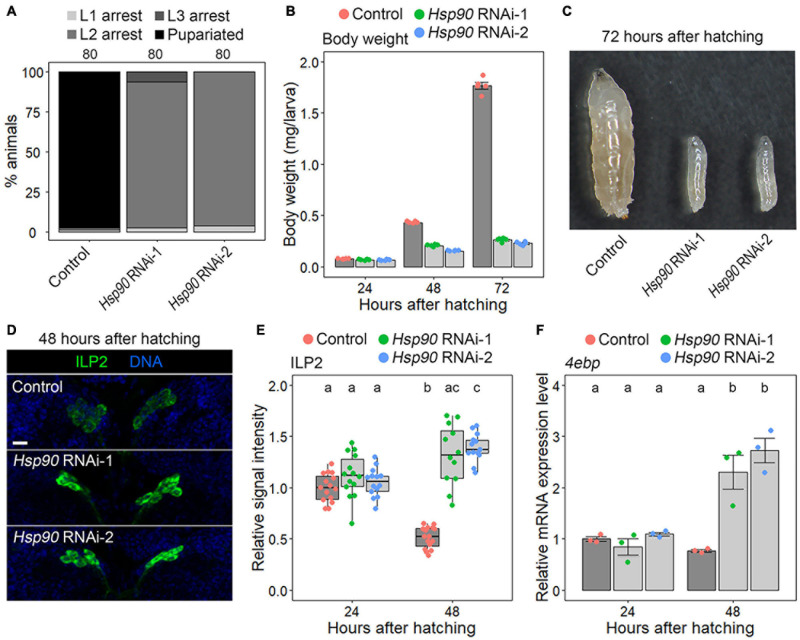
*Hsp90* is required for ILP release and systemic growth. **(A)** Fat-body-selective knockdown of *Hsp90* causes a developmental arrest at the 2nd instar larval stage. Percentages of pupariated animals (black) and arrested 1st instar (L1) (light gray)/2nd instar (L2) (gray)/3rd instar larvae (L3) (dark gray) in control (*Cg-Gal4* > +), *Hsp90 RNAi-1* (*Cg-Gal4* > *UAS-Hsp90 RNAi-1*), and *Hsp90 RNAi-2* (*Cg-Gal4* > *UAS-Hsp90 RNAi-2*) are shown. Sample sizes (the number of animals) are shown above each column. **(B)** Fat-body-selective knockdown of *Hsp90* causes a global growth defect. The body weight of control (red), *Hsp90 RNAi-1* (green), and *Hsp90 RNAi-2* animals (blue) is shown at indicated time points. Average values of five datasets with SE and scatter plots are shown. Ten to 15 larvae were pooled in each sample. **(C)** Control animals (left) developed into the 3rd instar larval stage and *Hsp90* RNAi-1 (middle) and RNAi-2 animals (right) arrested at the L2 stage. The photograph was taken at 72 h after hatching. **(D)** ILP2 immunostaining signal increased in the brains of fat-body-selective *Hsp90* knockdown animals. The brains of control (upper panel), *Hsp90* RNAi-1 (middle panel), and *Hsp90* RNAi-2 animals (lower panel) were labeled for ILP2 (green) and DNA (blue) at 48 h after hatching. Scale bar, 10 μm. **(E)** The ILP2 immunostaining signal intensities in the brains of control (red), *Hsp90 RNAi-1* (green), and *Hsp90 RNAi-2* animals (blue) are shown at indicated time points. The relative signal intensities are shown with box plots. The mean value of the controls at 24 h after hatching was set to 1. Sample sizes were 12–16. Different lowercase letters indicate statistically significant differences (*P* < 0.05; Steel–Dwass test). **(F)** The expression levels of *4ebp*, which were measured using qPCR, were elevated in fat-body-selective *Hsp90* knockdown animals. The relative expression levels of *4ebp* in control (red), *Hsp90 RNAi-1* (green), and *Hsp90 RNAi-2* animals (blue) are shown at indicated time points. Triplicate datasets of the *4ebp* expression levels are shown with average values, SEs, and scatter plots. Different lowercase letters indicate statistically significant differences (*P* < 0.05; Tukey’s test).

First, we confirmed that the expression level of GFP-fused Hsp90 (Hsp90::GFP), expressed under the control of endogenous *Hsp90* upstream sequences (Sarov et al., 2016), was reduced in the fat bodies of both *Hsp90* RNAi-1 and RNAi-2 animals (*Cg-Gal4* > *UAS-Hsp90 RNAi-1/2, Hsp90::GFP*) compared to that in control animals (*Cg-Gal4* > +, *Hsp90::GFP*) (Supplementary Figure 1). This result indicates that these RNAi constructs are effective in reducing *Hsp90* expression. Additionally, we confirmed that control animals (*Cg-Gal4* > +) developed from larval to pupal stages, whereas *Hsp90* RNAi-1 and RNAi-2 animals (*Cg-Gal4* > *UAS-Hsp90 RNAi-1/2*) were arrested at the second instar larval stage (Figure 2A). Next, we measured the body weight of control and *Hsp90* RNAi animals at 24, 48, and 72 hAH. At 24 hAH, the body weights of *Hsp90* RNAi-1 and RNAi-2 animals were equivalent to those of control animals (Figure 2B). The weights of the control larvae reached approximately 1.8 mg at 72 hAH (Figure 2B), whereas those of *Hsp90* RNAi-1 and RNAi-2 animals were approximately 0.25–0.3 mg at the same stage (Figures 2B,C). These results indicate that *Hsp90* expressed in the fat body is required for the proper progression of systemic growth from 24 hAH onward.

Since ILPs released from IPCs promote systemic growth, we assumed that ILP secretion and systemic insulin signaling are impaired in *Hsp90* knockdown animals. To test this possibility, the signal intensity of ILP2, the major ILP secreted from IPCs, was measured in control and *Hsp90* RNAi animals through immunostaining. If ILP2 secretion is blocked, ILP2 granules accumulate in IPCs, and hence the ILP immunostaining level is elevated. The signal intensity of ILP2 in *Hsp90* RNAi-1 and RNAi-2 animals was equivalent to that of control animals at 24 hAH (Figure 2E), but was significantly elevated in *Hsp90* RNAi-1 and RNAi-2 animals at 48 hAH (Figures 2D,E). This result indicates that IPC activity is suppressed in *Hsp90* knockdown animals at 48 hAH but not at 24 hAH. Furthermore, qPCR analysis revealed that the expression level of *eIF-4E-binding protein* (*4ebp*), which is suppressed by insulin signaling (Hietakangas and Cohen, 2009), was significantly increased in *Hsp90* knockdown animals at 48 hAH but not at 24 hAH (Figure 2F). Together, these results indicate that adipose *Hsp90* is required for ILP secretion and systemic insulin signaling from 24 hAH onward.

### *Hsp90* Is Required for Transcriptional Upregulation of Insulinotropic Signal Peptides

**FIGURE 3:**
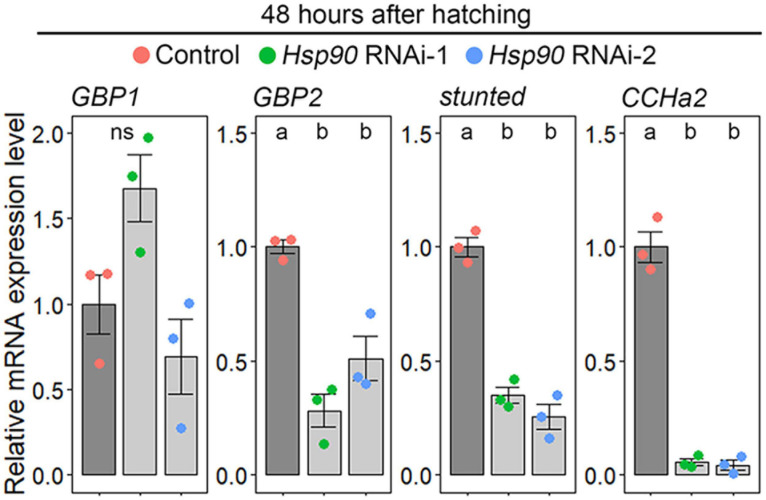
*Hsp90* promotes *GBP2*, *stunted*, and *CCHa2* expression in the fat body. The expression levels of *GBP2*, *stunted*, and *CCHa2* were reduced in the fat bodies of *Hsp90* knockdown animals. The expression levels of *GBP1*, *GBP2*, *stunted*, and *CCHa2* in the fat bodies of control (*Cg-Gal4* > +) (red), *Hsp90 RNAi-1* (*Cg-Gal4* > *UAS-Hsp90 RNAi-1*) (green), and *Hsp90 RNAi-2* (*Cg-Gal4* > *UAS-Hsp90 RNAi-2*) (blue) were measured by qPCR at 48 h after hatching. The average values of triplicate datasets are shown as SEs and scatter plots. Different lowercase letters indicate statistically significant differences (*P* < 0.05; Tukey’s test).

Because ILP secretion is remotely controlled by fat-body-derived insulinotropic/insulinostatic signal peptides, we used qPCR to investigate whether *Hsp90* knockdown causes downregulation of insulinotropic signal peptides or upregulation of insulinostatic signal peptides in the fat body. We selected *GBP1*, *GBP2*, *stunted*, and *CCHa2* as insulinotropic signal peptide-coding genes because they promote ILP2 secretion and growth (Sano et al., 2015; Delanoue et al., 2016; Koyama and Mirth, 2016; Meschi et al., 2019). *GBP1* expression was not affected in *Hsp90* RNAi-1 and 2, whereas *GBP2*, *stunted*, and *CCHa2* expression levels were significantly reduced in the fat bodies of *Hsp90* RNAi-1 and RNAi-2 animals (Figure 3). This indicates that *Hsp90* is required for the transcriptional upregulation of *GBP2*, *stunted*, and *CCHa2*. In contrast, we failed to amplify transcripts of TACE, which promotes secretion of the insulinostatic signal peptide Eiger (Agrawal et al., 2016), although we tested three independent primer sets (Supplementary Table 5). Thus, we could not determine whether *Hsp90* regulates *TACE* expression in the fat body.

### *Hsp90* Expression Is Dependent on Protein/Amino Acid Feeding

**FIGURE 4:**
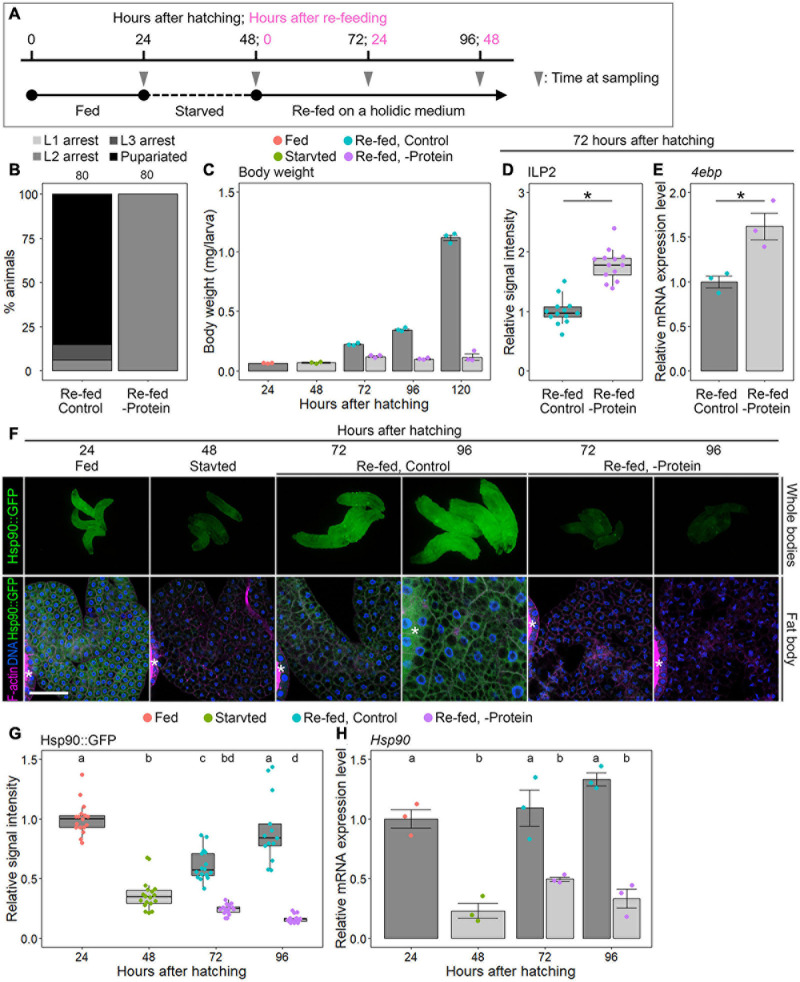
*Hsp90* expression is dependent on protein feeding. **(A)** Starvation and re-feeding scheme. **(B)** Protein feeding is required for larval-pupal transition. Percentages of pupariated animals (black) and arrested 1st instar (L1) (light gray)/2nd instar (L2) (gray)/3rd instar larvae (L3) (dark gray) in wild-type (WT, *Oregon R*) animals re-fed on a control or protein-deficient holidic medium are shown. Sample sizes (number of animals) are shown above each column. **(C)** Protein feeding is required for systemic growth. The body weights of WT larvae reared on a German medium (red), starved on a starvation medium (green), and re-fed on a control (light blue) or a protein-deficient holidic medium (purple) are shown at indicated time points. Triplicate datasets are shown with average values, SEs, and scatter plots. Ten to 15 larvae were pooled in each sample. **(D)** ILP2 immunostaining signal is elevated in the brain of WT larvae re-fed on a protein-deficient holidic medium. ILP2 immunostaining signal intensities in the brains of WT larvae re-fed on a control (light blue) or a protein-deficient holidic medium (purple) were measured at 72 h after hatching (24 h after re-feeding). The relative signal intensities are shown with box plots. The mean value of the controls was set to 1. Sample size was 13 in both groups. Asterisk indicates statistically significant differences (*P* < 0.01; Steel–Dwass test). **(E)** *4ebp* expression is elevated in WT larvae re-fed on a protein-deficient holidic medium. The relative expression levels of *4ebp* in WT larvae re-fed on a control (light blue) or a protein-deficient holidic medium (purple) were measured using qPCR at 72 h after hatching (24 h after re-feeding). Triplicate datasets of the *4ebp* expression levels are shown with average values, SEs, and scatter plots. Asterisk indicates statistically significant difference (*P* < 0.01; *t*-test). **(F)** GFP fluorescence is reduced in Hsp90::GFP-expressing larvae re-fed on a protein-deficient holidic medium. Hsp90::GFP-expressing larvae reared on a German medium (“Fed”), starved on a starvation medium (“Starved”), and re-fed on a control (“Re-fed, Control”) or a protein-deficient holidic medium (“Re-fed, -Protein”) were observed at indicated time points. Whole-bodies (upper panels) and fat bodies (lower panels) of Hsp90::GFP-expressing larvae are shown along with F-actin (magenta in lower panels) and DNA (blue in lower panels). Asterisks indicate the salivary gland. Scale bar, 50 μm. **(G)** The GFP signal intensities in the fat bodies of Hsp90::GFP-expressing animals reared on a German medium (red), starved on a starvation medium (green), and re-fed on a control (light blue) or a protein-deficient holidic medium (purple) are shown at indicated time points. The relative signal intensities of GFP are shown with box plots. The mean value at 24 h after hatching was set to 1. Sample sizes were 13–18. Different lowercase letters indicate statistically significant differences (*P* < 0.05; Steel–Dwass test). **(H)** *Hsp90* expression is downregulated in the fat bodies of WT larvae re-fed on a protein-deficient holidic medium. The *Hsp90* expression levels in the fat bodies of WT animals reared on a German medium (red), starved on a starvation medium (green), and re-fed on a control (light blue) or a protein-deficient holidic medium (purple) were measured by qPCR at indicated time points. The average values of triplicate datasets are shown with SEs and scatter plots. Different lowercase letters indicate statistically significant differences (*P* < 0.05; Tukey’s test).

Next, to test whether protein/amino acid availability affected *Hsp90* expression, we measured the *Hsp90* expression levels in the following starved and re-fed larvae: larvae reared on nutrient-rich German food were starved from 24 to 48 hAH; they were re-fed on holidic media with or without protein until 96 hAH (Figure 4A and Supplementary Table 4). *Hsp90* expression, developmental phenotype, and insulin signaling activity were examined using the WT (*Oregon R*) and Hsp90::GFP-expressing animals. First, we ascertained that WT larvae re-fed on a control holidic medium had an increased body weight and underwent pupariation; however, re-feeding WT animals on a protein-deficient holidic medium resulted in growth and developmental arrest (Figures 4B,C) and also caused an increase in the ILP2 immunostaining signal intensities and *4ebp* expression (Figures 4D,E). These data are consistent with previous studies showing the protein/amino acid dependency of ILP release and larval growth (Sang, 1956; Géminard et al., 2009). We then observed Hsp90::GFP expression in the whole body and fat body in this starvation/re-feeding experiment. We found that Hsp90::GFP signal intensity was reduced after 24-hour starvation and was restored in the whole body and fat body by control holidic medium feeding (Figures 4F,G). In contrast, Hsp90::GFP expression remained downregulated in larvae cultured on protein-deficient holidic media (Figures 4F,G). This indicates that Hsp90 expression depends on protein/amino acid availability. Likewise, *Hsp90* mRNA expression in the fat body was reduced by fasting and was restored by re-feeding of a protein-containing control holidic medium (Figure 4H). These results indicate that *Hsp90* expression is upregulated at the transcriptional level, depending on protein/amino acid availability.

### TORC1 Upregulates *Hsp90* Expression

**FIGURE 5:**
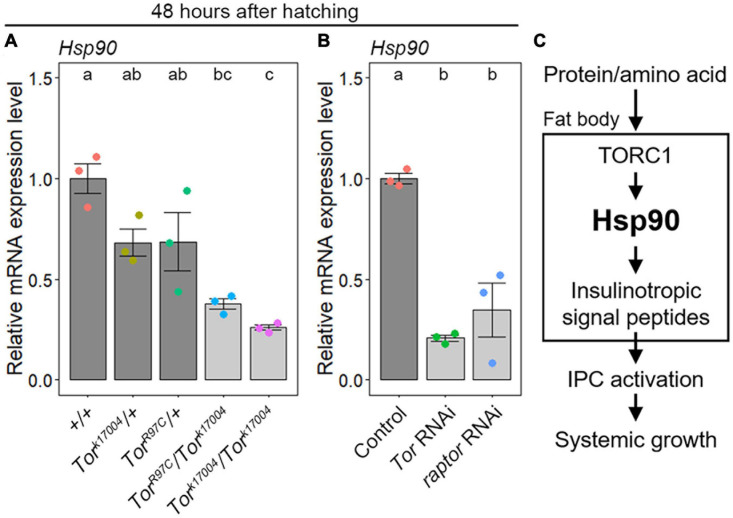
TORC1 signaling positively regulates *Hsp90* expression in the fat body. **(A)** Loss of *Tor* activity causes a reduction in *Hsp90* expression in the fat body. The *Hsp90* expression levels in the fat bodies of WT (+/+) (red), heterozygous *Tor* mutants (*Tor^*k*17004^*/+, *Tor^*R*97*C*^*/+) (yellow and green, respectively), homozygous *Tor^*k*17004^* mutants (*Tor^*k*17004^/Tor^*k*17004^*) (light blue), and transheterozygous *Tor* mutants (*Tor^*k*17004^/Tor^*R*97*C*^*) (purple) were measured using qPCR at 48 h after hatching. The average values of triplicate datasets are shown with SE and scatter plots. Different lowercase letters indicate statistically significant differences (*P* < 0.05; Tukey’s test). **(B)** Fat-body-selective *Tor* knockdown causes a reduction in *Hsp90* expression. The *Hsp90* expression levels in the fat bodies of the control (*Cg-Gal4* > +) (red), *Tor* RNAi (*Cg-Gal4* > *UAS-Tor RNAi*) (green), and *raptor* RNAi animals (*Cg-Gal4* > *UAS-raptor RNAi*) (blue) were measured using qPCR at 48 h after hatching. The average values of triplicate datasets are shown with SEs and scatter plots. Different lowercase letters indicate statistically significant differences (*P* < 0.05; Tukey’s test). **(C)** A current working model for systemic growth regulation by Hsp90. *Hsp90* expression is upregulated by TORC1-mediated protein/amino acid signaling. Adipocyte *Hsp90* promotes gene expression of insulinotropic signal peptides, and subsequently, *Hsp90* remotely promotes IPC activity and systemic growth.

Considering that TORC1 senses protein/amino acid availability, the above results raise the possibility that TORC1 controls *Hsp90* expression. To address this hypothesis, we investigated *Hsp90* expression levels in the fat bodies of *Tor* mutants and their developmental phenotypes. As shown in Supplementary Figure 2, *Tor^*k*17004^* and *Tor^*R*97*C*^* homozygous mutants (*Tor^*k*17004^/Tor^*k*17004^* and *Tor^*R*97*C*^/Tor^*R*97*C*^*) (Oldham et al., 2000; Zhang et al., 2000, 2006) showed developmental arrest at the second and first instar larval stages, respectively. In addition, transheterozygous *Tor* mutants (*Tor^*k*17004^/Tor^*R*97*C*^*) showed delayed pupariation. In contrast, WT (+/+) and heterozygous *Tor* mutants (*Tor^*k*17004^*/+ and *Tor^*R*97*C*^*/+) did not show any developmental defects. In accordance with the phenotypic severity, *Hsp90* expression was not significantly decreased in the fat bodies of heterozygous *Tor^*k*17004^* and *Tor^*R*97*C*^* mutants compared to WT, whereas it was significantly reduced in the fat bodies of *Tor^*k*17004^*/*Tor^*R*97*C*^* transheterozygous and *Tor^*k*17004^* homozygous mutants at 48 hAH (Figure 5A). These results indicate that *Tor* is required for the upregulation of *Hsp90* expression in the fat body. Furthermore, fat-body-selective knockdown of *Tor* (*Cg-Gal4* > *UAS-Tor RNAi*) and TORC1 component *raptor* (*Cg-Gal4* > *UAS-raptor RNAi*) caused a delay in pupariation (Supplementary Figure 2), and *Hsp90* transcript levels were reduced in the fat bodies of *Tor* and *raptor* knockdown animals (Figure 5B). These results indicate that TORC1 is required for the upregulation of *Hsp90* expression in the fat body. However, fat-body-selective *Hsp90* overexpression did not rescue the developmental delay in *Tor* RNAi animals (Supplementary Figure 2), suggesting that TORC1 likely regulates not only *Hsp90* but also other effectors to promote growth and development. Additionally, the signal intensity of the canonical TORC1 signaling marker, phospho-S6 (Romero-Pozuelo et al., 2017), was not significantly reduced in the fat bodies of *Hsp90* RNAi-1 and RNAi-2 animals (Supplementary Figure 3), suggesting that *Hsp90* has little effect on TORC1 signaling activity in the fat body.

We assumed that ambient temperature controls *Hsp90* expression since Hsp90 belongs to the HSP family, a group of heat-inducible molecular chaperones (Ashburner and Bonner, 1979). However, when Hsp90::GFP-expressing larvae were cultured at 29, 25, or 18°C, we observed from their histochemistry that temperature did not significantly affect Hsp90::GFP levels in the fat body (Supplementary Figure 4). This result suggests that *Hsp90* expression is robust, at least in this temperature range during larval development.

Taken together, these results indicate that *Hsp90* expression is upregulated by TORC1-mediated protein/amino acid signaling in the fat body (Figure 5C). In addition, considering that the expression of insulinotropic signal peptides, IPC activity, and growth rate were reduced in fat-body-selective *Hsp90* knockdown animals, it is reasonable to propose that adipose *Hsp90* remotely controls IPC activity and systemic growth through the regulation of insulinotropic signal peptides (Figure 5C).

## Discussion

### Novel Regulators of Fat-Body-Mediated Systemic Growth

In this study, we performed fat body-selective RNAi screening and identified *CG1965*, *CG16903*, *Ntf-2*, *Hsp90*, and *opm* as novel regulators of fat-body-mediated larval growth. Among these genes, *CG1965* and *CG16903* are functionally uncharacterized genes in *Drosophila*. *CG1965* encodes a protein possessing a domain named GC-rich sequence DNA-binding factor, and its amino acid sequence is orthologous to human PAX3 and PAX7 binding protein 1 (PAXBP1) (see FlyBase; FBgn0037466), which serves as an adaptor protein linking the histone methyltransferase complex to a transcription factor PAX7 and regulates epigenetic regulation of target genes (Diao et al., 2012). Interestingly, human *PAXBP1* possesses a potential pathogenic genetic variation in familial growth defects (Alharby et al., 2017). Furthermore, *cyclin L1*, a human ortholog of *CG16903* (FBgn0040394), has been reported to be associated with fetal growth and birth weight (Freathy et al., 2010). Cyclin L1 participates in the regulation of RNA polymerase II transcription regulation and pre-mRNA processing (Dickinson et al., 2002). The role of *CG1965* and *CG16903* in *Drosophila* development is unclear; however, the above observations suggest the possibility that *CG1965* and *CG16903* encode evolutionarily conserved growth regulators, and *Drosophila* may serve as a useful genetic model to study the *in vivo* functions of these genes.

### Transcriptional Regulation of *Hsp90* Expression

We found that *Hsp90* expression is dependent on protein/amino acid feeding, and TORC1 upregulates its transcription (Figures 4, 5). Considering that TORC1 positively regulates the transcription factor Myc in *Drosophila* (Teleman et al., 2008; Demontis and Perrimon, 2009; Li et al., 2010; Parisi et al., 2011) and that Myc directly activates *Hsp90* transcription in mammals (Teng et al., 2004), Myc is a potential transcriptional regulator of *Hsp90* in *Drosophila*. It has also been reported that TOR upregulates the transcription factor DNA replication-related element-binding factor (DREF) partially via Myc in the fat body (Killip and Grewal, 2012). These findings and our data lead to the hypothesis that TORC1 regulates *Hsp90* transcription through Myc and DREF. Alternatively, because *Hsp90* and other *Hsp* genes are induced by a transcription factor called heat shock factor (HSF) upon induction of heat shock and other environmental stresses (Gomez-Pastor et al., 2018), it is also possible that HSF induces *Hsp90* transcription in a TORC1-dependent manner, although it remains unclear whether HSF activity is dependent on proteins/amino acids and TORC1. It will be interesting to know whether HSF, Myc, DREF, or other transcriptional regulators upregulate *Hsp90* expression in response to TORC1-mediated nutrient signals.

### TORC1- and Hsp90-Mediated Regulation of Insulinotropic Signal Peptides in the Fat Body

Chaperones generally support unspecific protein folding; however, Hsp90 seems to be less promiscuous than other chaperones (Wandinger et al., 2008). Hsp90 binds to client proteins such as transcription factors and signaling proteins in a near-native state at a later stage of folding to potentiate their functions (Pratt and Toft, 1997; Young et al., 2001; Wandinger et al., 2008; Trepel et al., 2010). Hsp90 is involved in various biological processes, including gene expression, chromatin remodeling, cell cycle progression, stem cell reactivation, and exosome release (Pratt and Toft, 1997; Tariq et al., 2009; Trepel et al., 2010; Sawarkar et al., 2012; Bandura et al., 2013; Huang and Wang, 2018; Lauwers et al., 2018). We revealed that *Hsp90* promotes ILP2 secretion and systemic growth progression (Figure 2). Furthermore, we found that *GBP2*, *stunted*, and *CCHa2* expression was reduced in the fat body of *Hsp90* RNAi animals (Figure 3), indicating that *Hsp90* upregulates the expression of these insulinotropic signal peptides. Previous studies have shown that *GBP2* and *CCHa2* expression is dependent on protein/amino acid feeding and TORC1 signaling (Sano et al., 2015; Koyama and Mirth, 2016). Hence, it is likely that TORC1 upregulates *GBP2* and *CCHa2* transcription via Hsp90. In contrast, the expression of *stunted* is regulated by the transcription factor peroxisome proliferator activated receptor-γ coactivator-1 (PGC1)/Spargel, whereas Stunted secretion into the hemolymph is governed by TORC1 (Delanoue et al., 2016). Thus, it is probable that *Hsp90* interacts with PGC1 to regulate *stunted* expression. In contrast to *GBP2*, *stunted*, and *CCHa2*, *GBP1* expression levels were not affected in *Hsp90* RNAi animals (Figure 3), although *GBP1* expression is also promoted by protein/amino acid feeding and TORC1 signaling (Koyama and Mirth, 2016). Therefore, it is likely that *GBP1* transcription is regulated by Hsp90-independent but TORC1-dependent pathways. Indeed, fat-body-selective *Hsp90* overexpression did not rescue the developmental delay in *Tor* RNAi animals (Supplementary Figure 2). Thus, TORC1 likely regulates not only *Hsp90* but also other effectors to activate the insulinotropic signal peptide expression and systemic growth.

Because the fat body also secretes the insulinostatic signal peptide Eiger to downregulate ILP production in response to nutrient starvation (Agrawal et al., 2016), one possible mechanism is that Hsp90 suppresses the expression of TACE, a rate-limiting factor for the secretion of Eiger under nutrient-rich conditions. However, we failed to amplify *TACE* transcripts using qPCR. Thus, *TACE* transcription might be active in a narrow time window, or its expression pattern is influenced by unknown environmental conditions. Further studies are required to elucidate the transcriptional regulatory mechanisms of fat-body-derived signal peptides.

Another possible function of Hsp90 is to stabilize fat-body-derived peptides so that they can travel a large distance to the CNS. In mammals, Hsp90 is secreted into the extracellular space (Eustace et al., 2004; Xiaofeng et al., 2009). Considering that signal peptides such as EGF, including GBPs, only act locally, whereas fat-body-derived GBPs can reach the CNS as peptide hormones (Miura et al., 2006; Koyama and Mirth, 2016; Meschi et al., 2019), it is possible that the fat body secretes Hsp90 along with signal peptides to increase their stability. Moreover, recent studies have revealed that Hsp90 directly binds and remodels plasma membranes to promote the fusion of multivesicular bodies with the cell membrane and the subsequent release of exosomes in the *Drosophila* nervous system (Lauwers et al., 2018). Further studies are necessary to elucidate the role of Hsp90 in the adipocyte secretory system.

In conclusion, adipose *Hsp90* acts as a nutrient-responding factor and promotes growth and development, at least in part, via the fat body-IPC axis. To our knowledge, this is the first study to show that *Hsp90* serves as a nutrient-responsive growth regulator. Because the *Hsp90* expression level seems to be dependent on proteins/amino acid feeding in many larval tissues (Figure 4), *Hsp90* likely functions ubiquitously in response to protein/amino acids during larval development. Considering that juvenile animals need to build up their bodies by continuous intake of available macronutrients such as amino acids and proteins, we propose that *Hsp90* is upregulated under such “amino acid/protein-rich stress” conditions to support continuous and rapid protein anabolism and growth.

## Data Availability Statement

The original contributions presented in the study are included in the article/Supplementary Material, further inquiries can be directed to the corresponding author/s.

## Author Contributions

YO and KY-K contributed to conception and design of the study. YO performed the statistical analysis and wrote the first draft of the manuscript. All authors contributed to experiment, manuscript revision, read, and approved the submitted version.

## Conflict of Interest

The authors declare that the research was conducted in the absence of any commercial or financial relationships that could be construed as a potential conflict of interest.

## Footnotes

1 http://www.R-project.org/
