# Supplementary material for: The Nutrient-Responsive Molecular Chaperone Hsp90 Supports Growth and Development in Drosophila
Source: Front Physiol. 2021 Jun 22;12:690564. doi: 10.3389/fphys.2021.690564 (PMC8258382; doi:10.3389/fphys.2021.690564)
Supplement: Supplementary file 7 [file Data_Sheet_1.docx]

***Supporting Information***

**Figure S1. The Hsp90::GFP expression level in the fat bodies of *Hsp90* RNAi animals**

**(A)** Hsp90::GFP expression is reduced in the fat bodies of *Hsp90* knockdown animals. The Hsp90::GFP expression in the fat bodies of control (*Cg-Gal4 > +, Hsp90::GFP*) (left panel), *Hsp90* RNAi-1 (*Cg-Gal4 > UAS-Hsp90 RNAi-1, Hsp90::GFP*) (middle panel), and *Hsp90* RNAi-2 animals (*Cg-Gal4 > UAS-Hsp90 RNAi-2, Hsp90::GFP*) (right panel) was observed at 48 h after hatching. Hsp90::GFP (green) is shown along with F-actin (magenta) and DNA (blue). Scale bar, 10 μm. **(B)** The GFP signal intensities in the fat bodies of control (red), *Hsp90* RNAi-1 (green), and *Hsp90* RNAi-2 animals (blue) are shown with average values, SEs, and scatter plots. Sample sizes in the control, *Hsp90* RNAi-1, and *Hsp90* RNAi-2 were 13, 18, and 15, respectively. Different lowercase letters indicate statistically significant difference (*P* < 0.05; Tukey’s test).

**Figure S2. The developmental profiles of *Tor* mutants and knockdown animals**

**(A)** Percentages of pupariated animals (black) and arrested 1st instar (L1) (light gray)/2nd instar (L2) (gray)/3rd instar larvae (L3) (dark gray) in WT (*+/+*), heterozygous *Tor* mutants (*Tor^k17004^/+*, *Tor^R97C^/+*), homozygous *Tor* mutants (*Tor^k17004^/Tor^k17004^, Tor^R97C^/Tor^R97C^*), and transheterozygous *Tor* mutants (*Tor^k17004^/Tor^R97C^*) are shown. Sample sizes (the number of animals) are shown beside each column. **(B)** Developmental timing is delayed in *Tor^k17004^/Tor^R97C^* mutant. Percentages of pupariated animals in WT (red), heterozygous *Tor* mutants (*Tor^k17004^/+*, *Tor^R97C^/+*) (yellow and green, respectively), and transheterozygous *Tor^k17004^/Tor^R97C^* mutant (light blue) are shown at indicated time points. **(C)** Percentages of pupariated animals (black) and arrested 1st instar (L1) (light gray)/2nd instar (L2) (gray)/3rd instar larvae (L3) (dark gray) in control (*Cg-Gal4 > +*), *Tor* RNAi (*Cg-Gal4 > UAS-Tor RNAi*), and *raptor* RNAi animals (*Cg-Gal4 > UAS-raptor RNAi*) are shown. Sample sizes (the number of animals) are shown beside each column. **(D)** Developmental timing is delayed in fat-body-selective *Tor* knockdown animals. Percentages of pupariated animals in control (red), *Tor* RNAi (green), and *raptor* RNAi animals (blue) are shown at indicated time points. **(E)** Overexpression of *Hsp90* does not rescue a delay in pupariation in *Tor* knockdown animals. Percentages of pupariated animals in *Tor* RNAi (green) and *Hsp90*-overexpressing *Tor* RNAi animals (*Cg-Gal4 > UAS-Tor RNAi UAS-Hsp90*) (blue) are shown at indicated time points. Sample sizes (the number of animals) were 95 and 70, respectively.

**Figure S3. The phospho-S6 expression level in the fat bodies of *Hsp90* RNAi animals**

**(A)** The expression levels of phospho-S6, the canonical TORC1 signaling marker, are not reduced in the fat bodies of *Hsp90* RNAi animals. The fat bodies of control (*Cg-Gal4 > +*) (left panel), *Hsp90* RNAi-1 (*Cg-Gal4 > UAS-Hsp90 RNAi-1*) (middle panel), and *Hsp90* RNAi-2 animals (*Cg-Gal4 > UAS-Hsp90 RNAi-2*) (right panel) were labelled for phospho-S6 (green) and DNA (blue) at 48 h after hatching. Scale bar, 10 μm. **(B)** The phospho-S6 immunostaining signal intensities in the fat bodies of control (red), *Hsp90* RNAi-1 (green), and *Hsp90* RNAi-2 (blue) are shown with average values, SEs, and scatter plots. Sample sizes in control, *Hsp90* RNAi-1, and *Hsp90* RNAi-2 were 15, 12, and 11, respectively. Different lowercase letters indicate statistically significant difference (*P* < 0.05; Tukey’s test).

**Figure S4. The Hsp90::GFP expression levels in the fat bodies at 18, 25, or 29ºC**

**(A)** Temperature does not affect Hsp90::GFP expression in the fat body. The fat bodies of Hsp90::GFP-expressing animals at 18 (left panel), 25 (middle panel), or 29ºC (right panel) at 48 hAH are shown. Hsp90::GFP (green) is shown along with F-actin (magenta) and DNA (blue). Asterisks indicate the salivary glands. Scale bar, 50 μm. **(B)** The GFP signal intensities in the fat bodies of Hsp90::GFP-expressing animals reared at 18 (red), 25 (green), or 29ºC (blue) are shown at indicated time points. The relative signal intensities are shown with box plots, and the mean value at 25ºC at 24 hours after hatching was set to 1. Sample sizes are 13−18. Different lowercase letters indicate statistically significant difference (*P* < 0.05; Steel Dwass test).

**Table S1. Fly stocks used in this study**

^*^ BDSC, Bloomington *Drosophila* Stock Center; VDRC, Vienna *Drosophila* RNAi Center

**Table S2. Target genes, RNAi stocks, and their phenotypes in the 1st screening**

Virgins carrying homozygous *Cg-Gal4* (fat-body-selective Gal4) were crossed with *UAS-RNAi* males to obtain the offspring in which a target gene was knocked down in the fat body (*Cg-Gal4 > UAS-RNAi against gene-of-interest*). Note that all RNAi stocks were obtained from National Institute of Genetics, Japan. NOP, non-obvious phenotype.

**Table S3. Target genes, RNAi stocks, and their phenotypes in the 2nd screening**

Virgins carrying homozygous *Cg-Gal4* (fat-body-selective Gal4) were crossed with *UAS-RNAi* males to obtain the offspring in which a target gene was knocked down in the fat body (*Cg-Gal4 > UAS-RNAi against gene-of-interest*). ^*^ NIG, National Institute of Genetics (Japan); VDRC, Vienna *Drosophila* RNAi Center; BDSC, Bloomington *Drosophila* Stock Center. NOP, non-obvious phenotype.

**Table S4. Diet ingredient composition of holidic media**

**Table S5. The primer sets used for qPCR**
