# Supplementary figures and images for: The Nutrient-Responsive Molecular Chaperone Hsp90 Supports Growth and Development in Drosophila
Source: Front Physiol. 2021 Jun 22;12:690564. doi: 10.3389/fphys.2021.690564 (PMC8258382; doi:10.3389/fphys.2021.690564)

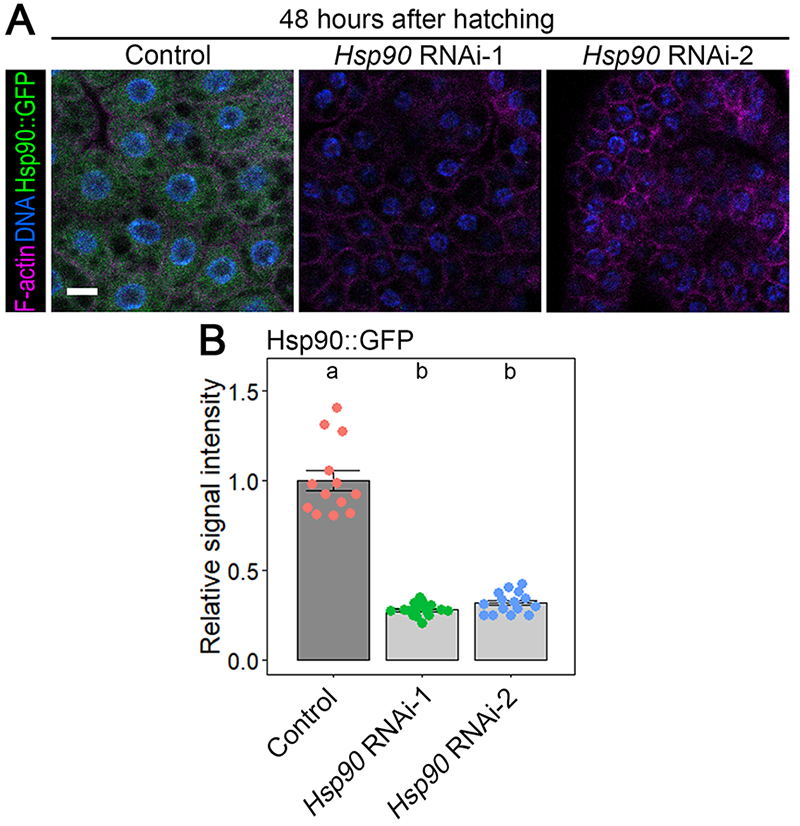

Supplement: Supplementary file 8 [file Image_1.TIF]

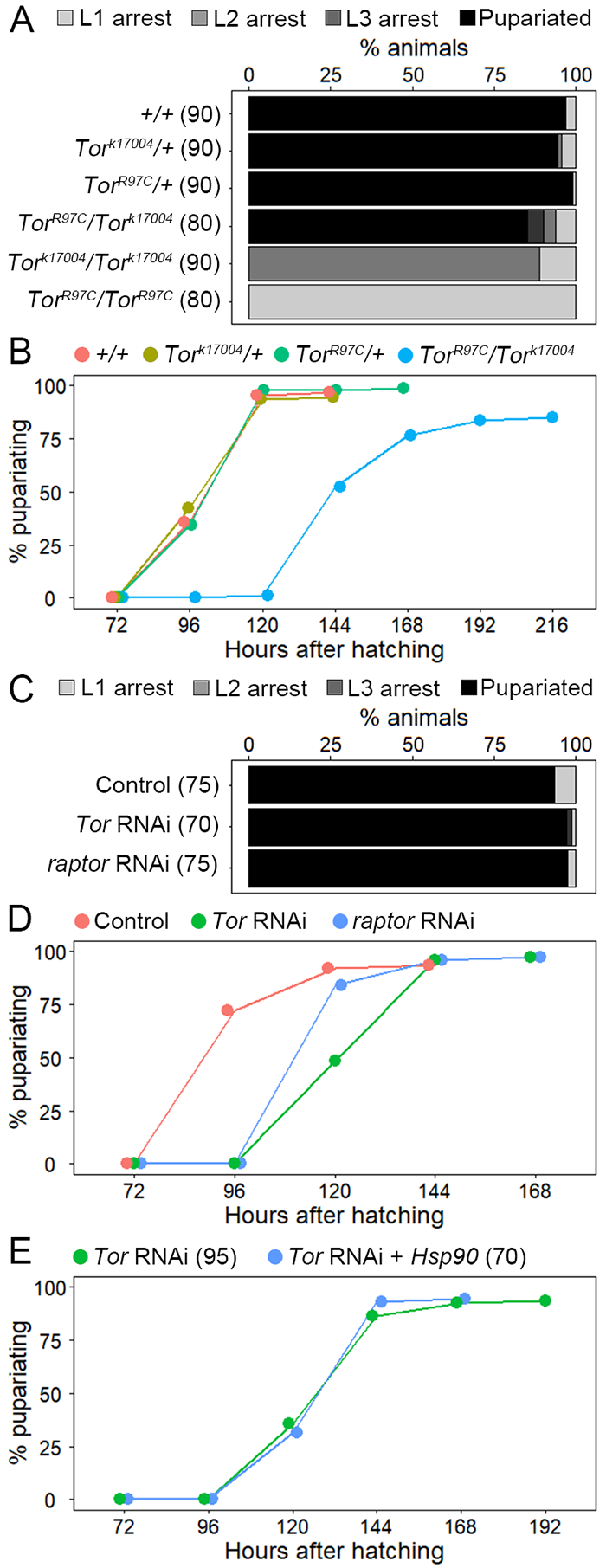

Supplement: Supplementary file 9 [file Image_2.TIF]

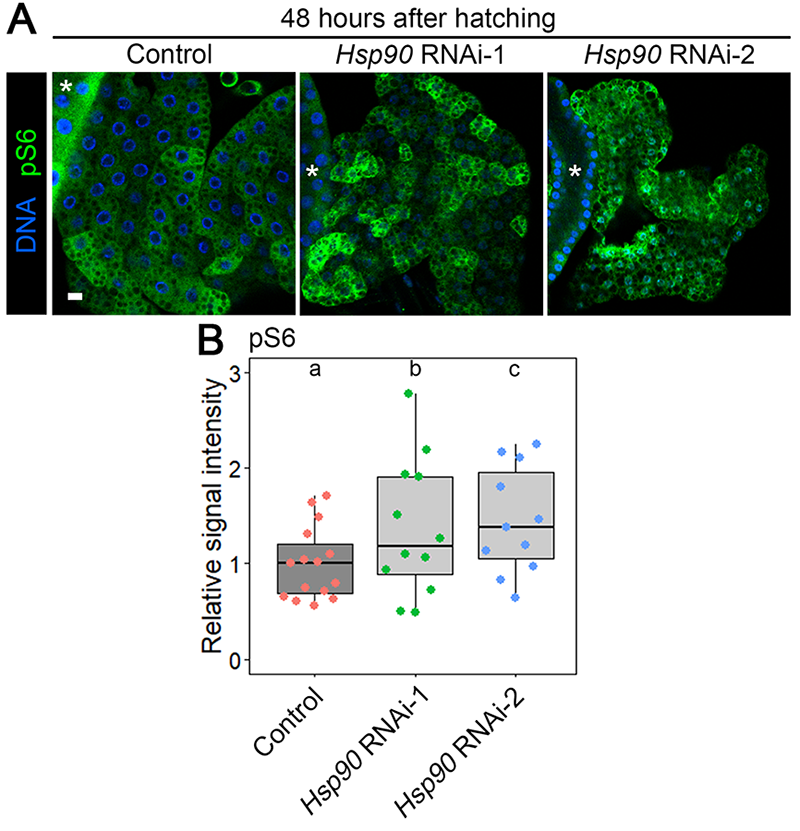

Supplement: Supplementary file 10 [file Image_3.TIF]

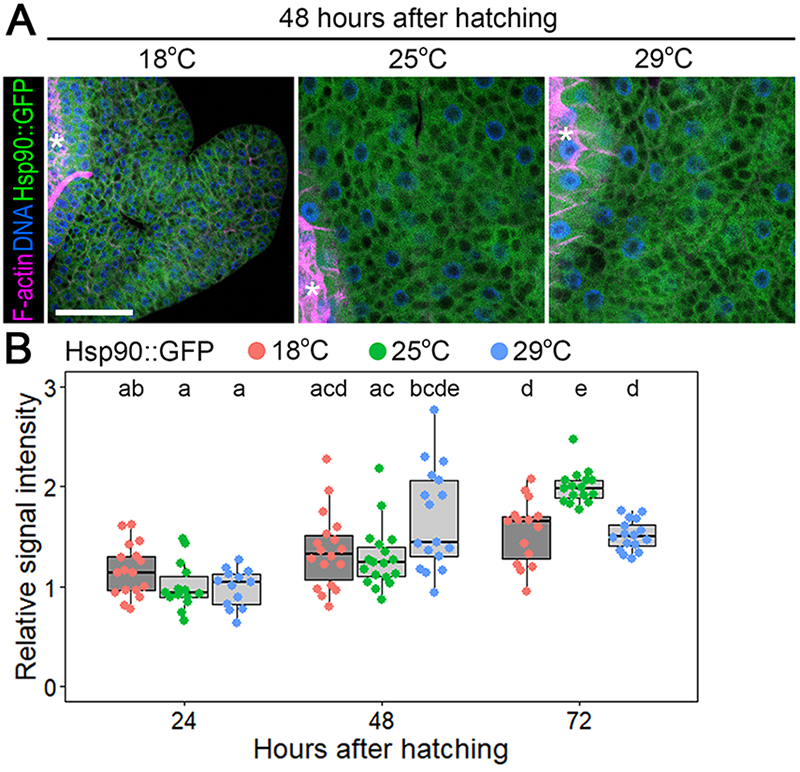

Supplement: Supplementary file 11 [file Image_4.TIF]
